# Emergency Care, Hospitalization Rates, and Floods

**DOI:** 10.1001/jamanetworkopen.2025.0371

**Published:** 2025-03-10

**Authors:** Zachary S. Wettstein, Canada Parrish, Amber K. Sabbatini, Matthew H. Rogers, Edmund Seto, Jeremy J. Hess

**Affiliations:** 1 Department of Emergency Medicine, School of Medicine, University of Washington, Seattle; 2 Climate Impacts Group, University of Washington, Seattle; 3 Department of Environmental and Occupational Health Sciences, School of Public Health, University of Washington, Seattle; 4 Department of Global Health, Schools of Medicine and Public Health, University of Washington, Seattle

## Abstract

**Importance:**

Flooding is a major environmental hazard, with events increasing in intensity and frequency in the context of climate change. Floods cause significant health and economic impacts, particularly among vulnerable populations, including older adults. However, comprehensive analyses of the health consequences of flooding remain limited.

**Objective:**

To evaluate the morbidity and health care costs among Medicare beneficiaries associated with flood exposure in the US.

**Design, Setting, and Participants:**

This retrospective cohort study analyzed emergency department (ED) use and unplanned hospitalization among Medicare beneficiaries 65 years or older living in zip code tabulation areas (ZCTAs) that were exposed to large-scale flood events from January 1, 2008, to December 31, 2017. This analysis was conducted from April 3 to December 15, 2023.

**Exposure:**

The primary exposure was the presence of a flood as recorded in the Multisourced Flood Inventories, a spatially distributed flood database.

**Main Outcomes and Measures:**

A conditional fixed-effects regression approach was used to explore the incidence of all-case and cause-specific ED visits and hospitalizations before and after floods. The primary outcomes measured were the incident rate ratios (IRRs) and associated 95% CIs. Attributable risk percentages and estimated attributable excess visits were calculated. Stratified analyses were performed for evaluation of effect modification. Health care costs associated with these events were measured and standardized to 2017 US dollars.

**Results:**

Among 11 801 527 Medicare beneficiaries 65 years or older (mean [SD] age, 74.4 [7.6] years; 56.3% female), the rate of all-cause ED visits and hospital admissions increased by 4.8% (IRR, 1.05; 95% CI, 1.04-1.05) and 7.4% (IRR, 1.07; 95% CI, 1.07-1.08) after flood exposure, respectively. The mean ZCTA-level cost was $3230 (95% CI, $3198-$3261) per ED visit and $11 310 (95% CI, $11 252-$11 367) for hospitalizations. The national costs to the Medicare system were estimated to be $69 275 429 (95% CI, $63 010 840-$76 315 210) for ED visits and $191 409 579 (95% CI, $172 782 870-$206 181 300) for hospitalizations. Stratified analyses highlighted greater impacts for certain demographic groups, including adults older than 85 years, and specific seasonal patterns.

**Conclusions and Relevance:**

In this cohort study of Medicare beneficiaries 65 years or older, flood exposure was associated with increased health care use and costs, underscoring the need for targeted public health strategies and improved disaster preparedness, especially for older adults. These findings contribute to a more comprehensive understanding of the health-related costs of flooding and can be used to inform future climate change resilience and health care planning.

## Key Points

**Question:**

Is there an association between flood exposure and health care use and costs among older Medicare beneficiaries in the US?

**Findings:**

This cohort study of more than 11 million Medicare beneficiaries 65 years or older found that flood exposure was associated with increased rates of emergency department visits and hospitalizations, particularly for conditions such as metabolic and kidney disorders, infectious diseases, and injuries, along with substantial health care costs.

**Meaning:**

These findings highlight the health and economic burden of flooding in the US and underscore the need for disaster preparedness and health care system planning to mitigate these impacts on vulnerable populations.

## Teaser

This cohort study examines the association between large-scale flood events and health care use among Medicare beneficiaries 65 years or older.

## Introduction

Flooding is the most common environmental hazard in the world, accounting for approximately 40% of all global natural disasters and up to 50% of all deaths due to natural disasters.^1,2,3^ The frequency and intensity of extreme precipitation events have increased over the past century and are expected to increase further with climate change, which has resulted in more large-scale flooding events.^4^ Flooding affects health via a myriad of pathways, including drowning, injuries related to debris and chemical contaminants, electrical injuries, and hypothermia.^5,6,7^ Infrastructure damage may lead to contaminated water and the spread of infectious diseases, disruptions of health services, and human displacement.^7,8^ These disruptions may lead to further downstream consequences, such as exacerbations of chronic disease and mental health conditions. Effects of flooding are not evenly distributed: during flood events, females, children, and older adults are at greatest risk of psychological and physical health effects, whereas young men are more likely to experience mortality.^2,9^ Older adults are likely more vulnerable to the health effects of flooding due to preexisting health conditions and compounded by the predisposition for mobility concerns diminishing their ability to respond to flood warnings and evacuations.^9,10^ Moreover, floods are among the costliest disasters in the US, many resulting in more than $1 billion in damages, with most of these extreme flooding events occurring in the past decade alone.^11,12^

Efforts to quantify the health impacts of large-scale flooding events in the US have been limited and constrained by underreporting of nonfatal injuries and health care use.^2^ Recent efforts have identified substantial health care burdens associated with tropical storms and hurricanes and localized impacts from flooding, although no nationwide evaluation has been published to date.^13,14,15^ Limited understanding of the consequences of flooding on health care use and associated health care costs limits health impact assessment, risk communication, estimation of climate change–related damages, and projection of climate change health impacts, all of which are needed to clarify climate change risks to human health and to increase resilience to climate-related flooding events through disaster risk reduction efforts.^16,17,18,19,20^

To address these gaps in our understanding of the health impacts of flooding and inform future public health preparedness in an era of increasing hydrological variability and climate change, we conducted a comprehensive analysis of the association between large-scale flood events and health care use among Medicare beneficiaries 65 years or older in the US. Using a decade-long flood dataset, we examined the incidence of emergency department (ED) visits and hospital admissions in the wake of flooding, alongside an assessment of associated health care costs, exploring potential variations across demographic groups and flood-related factors.

## Methods

### Study Design

This retrospective cohort study used administrative data from the continental US from January 1, 2008, through December 31, 2017. This study was determined to be exempt from review and the need for informed consent by the University of Washington Institutional Review Board because of the use of deidentified data. This study was conducted and reported in accordance with the Strengthening the Reporting of Observational Studies in Epidemiology (STROBE) reporting guideline.

Given variability in the lags between flood exposure and various health impacts, we first conducted several preliminary analyses to determine the most appropriate baseline and postflood intervals when we would expect to see changes in hospital use as a result of the flooding (eAppendix in Supplement 1). Using a series of interrupted time series models, we explored 4 different potential hazard periods, including 2, 4, 6, and 8 weeks before and after exposure. For our final analysis, we selected a 4-week flood hazard period starting on the date of flood onset and compared outcomes with a control period consisting of the 4 weeks before the start of the flood. This period was selected based on findings from the preliminary analysis and the use of a similar hazard period in other studies and to minimize the potential for type I error by extending the hazard period beyond biological plausibility.^13,15^ Outcomes of interest were assessed at the population level for beneficiaries living in zip code tabulation areas (ZCTAs) experiencing major flood events during the study window for the flood hazard period relative to outcomes for the same ZCTA in the control period.

### Study Population and Data Sources

Study participants were Medicare beneficiaries 65 years and older living in ZCTAs experiencing floods during the study period. Medicare beneficiaries younger than 65 years were excluded due to small sample size and the unique eligibility criteria for this age group.^21^

Flood exposure assessment was determined using the Multisourced Flood Inventories, a database of flood events in the continental US from 1998 to 2019.^22,23^ The Multisourced Flood Inventories database aggregates data from stream gauge observations, remote sensing, and model simulation to generate the most comprehensive, open-access database of floods currently available. Floods in the database exceeded a 20-year return period, meaning the mean number of years between a flood of this magnitude for this location was at least 20 years. The spatial resolution of the flood data was on the subcatchment level, a hydrological unit of land with a natural boundary where all surface water drains to a common channel or point. We converted subcatchment data to ZCTA-level floods for linkage with the Medicare beneficiary data. Flood extent was estimated by calculating the maximum surface area of contiguous ZCTAs flooded in the same event. Flood start date and the flooded ZCTAs were used to identify the spatiotemporal extent of the flood, and Medicare beneficiary home addresses were used to attribute flood exposure to individual beneficiaries.

Medicare beneficiary data were sourced from the Centers for Medicare & Medicaid Services (CMS), providing individual-level health care encounters, including discharge diagnoses, cost of claims, and demographic data. Individual race and ethnicity data were reported by the CMS and not modified by the research team and included the following categories: American Indian or Alaska Native, Asian, Black, Hispanic, White, other, and unknown. Data on race and ethnicity were collected to assess potential disparities in health care use and outcomes after flood exposure. Discharge diagnoses for ED visits and hospital admissions, categorized into cause-specific and all-cause diagnoses, were used for outcomes (eTable 1 in Supplement 1). Costs associated with these Medicare claims were tabulated on the 4-week flood hazard and control periods and used for the analysis of health care costs, standardized to 2017 US dollars. The CMS data used in this study have very low rates of missing data, and no specific interventions or imputation methods were necessary to address missingness.

The Centers for Disease Control and Prevention county-level Social Vulnerability Index (SVI) was incorporated as an additional covariate in the stratified analysis. County-level composite SVI values were acquired from the Centers for Disease Control and Prevention, and corresponding ZCTA-level composite SVI values were used to calculate SVI quartiles for the stratified analysis.

### Statistical Analysis

We conducted a ZCTA-level analysis of total all-cause and cause-specific diagnostic categories for ED visits and hospitalizations of flood-exposed cohorts (eTable 1 in Supplement 1). We performed a fixed-effects conditional negative binomial regression using total counts of all-cause and cause-specific encounters during the preflood and postflood periods to estimate incident rate ratios (IRRs) and 95% CIs, conditioning on the ZCTA. Model terms included flood exposure and a log-offset of the Medicare beneficiary population within a given ZCTA. This model selection was well suited to account for overdispersion often encountered in observed count data. Given that preliminary testing indicated overdispersion in our data, a negative binomial regression was used. Model fit was assessed through evaluating the association between raw-scale estimates and residuals and a nonsignificant Pearson correlation test.

To evaluate for effect modification by individual-level factors, a stratified analysis was conducted on sex, age category (65-74 years, 75-84 years, and ≥85 years), race (Black and White), flood season (winter, spring, summer, and fall), extent of area flooded (quartile), and composite SVI (quartile) to evaluate for potential effect modification. The stratified analysis among racial categories was limited to Black- and White-identified groups due to the small sample size among the other groups. Individual-level factors were aggregated on the ZCTA level to perform the stratified analysis. The attributable risk percentage for each outcome type was calculated based on the IRR, and the number of excess visits attributable to flood exposure was calculated by multiplying the attributable risk percentage by the number of specific visits in the flood period. These estimates were calculated for the 4-week hazard and control periods.

To assess the potential economic impact of large-scale flood events, we estimated the costs associated with incremental increases in ED and hospital use. Medicare claims include total payments made to hospitals for an ED visit or hospital stay. We calculated the mean payment for an ED visit or inpatient admission using total payments for each encounter in our dataset and then multiplied that by the number of excess visits or hospitalizations previously calculated to estimate the incremental increase in health care spending associated with flooding events. All costs were standardized to 2017 US dollars. To consider the financial impact systemwide, ZCTA-level costs of ED visits and hospitalizations were calculated and multiplied by the estimated number of excess visits by type. All analyses were performed using SAS software, version 9.4 (SAS Institute Inc) and Stata software, version 16.1 (StataCorp LLC). This analysis was conducted from April 3 to December 15, 2023.

## Results

### Flood Exposure Data

A total of 11 801 527 Medicare beneficiaries 65 years or older were included in the cohort (mean [SD] age, 74.4 [7.6] years; 56.3% female and 43.7% male; 0.5% American Indian or Alaska Native, 0.9% Asian, 7.5% Black, 1.2% Hispanic, 88.3% White, 1.0% other [no additional definition of “other” provided by CMS], and 0.7% unknown) (Table 1). The mean (SD) ZCTA flood exposure per beneficiary was 1.3 (0.6). From 2008 through 2017, there were 16 536 flooded ZCTAs among a total of 33 140. The median number of floods experienced by a flooded ZCTA was 1 (IQR, 1-2), and the median ZCTA flood duration was 10 (IQR, 4-30) days. The median area flooded was 34 769 (IQR, 17 319-65 584) square miles. The median number of Medicare beneficiaries per flooded ZCTA was 4142 (IQR, 2006-7536).

**Table 1.** Medicare Beneficiary Cohort Demographic Characteristics and Flood Exposure

Characteristic	Medicare beneficiaries, No. (%)^a^ (N = 11 801 527)
Age, mean (SD), y	74.4 (7.6)
Sex	
Male	5 156 174 (43.7)
Female	6 645 353 (56.3)
Race	
American Indian or Alaskan Native	56 147 (0.5)
Asian	101 190 (0.9)
Black	884 380 (7.5)
Hispanic	139 734 (1.2)
White	10 422 915 (88.3)
Other^b^	113 179 (1.0)
Unknown	83 982 (0.7)
No. of floods per beneficiary, mean (SD)	1.3 (0.6)

^a^ Unless otherwise indicated.

^b^ No additional information from the Centers for Medicare & Medicaid Services on “other” race.

There was monthly and annual variability in flood frequency and duration (eTables 2 and 3 in Supplement 1). Floods were most frequent in May (3628 ZCTA floods) and least frequent in November (112 ZCTA floods). The greatest number of floods occurred in 2015 (3108 ZCTA floods), whereas the fewest floods occurred in 2009 (942 ZCTA floods), with a median (IQR) duration ranging from 5 (4-10) days (in 2010) to 35 (7-35) days (in 2014).

**Figure 1.**
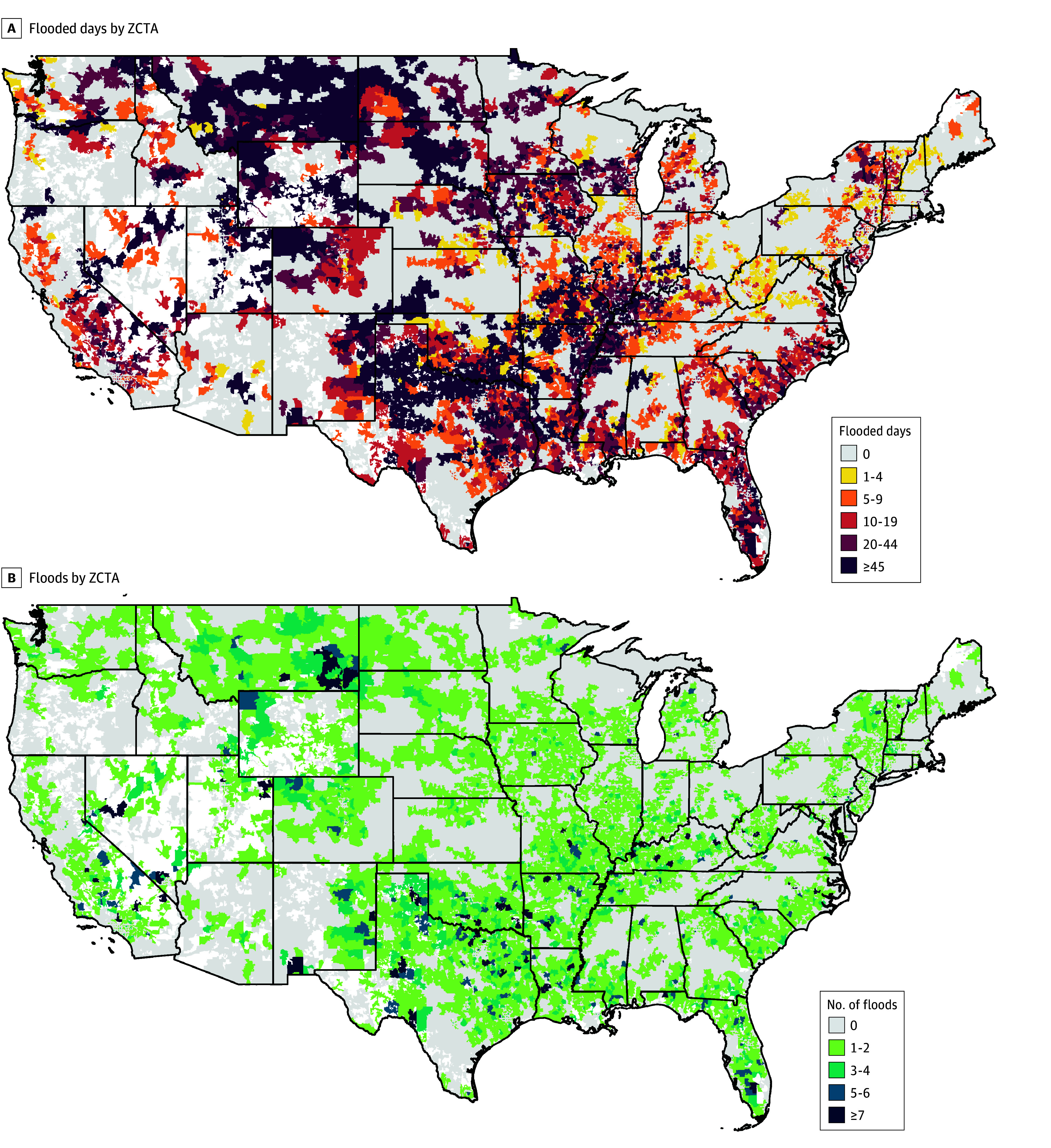
Total Number of Floods and Quintile of the Total Duration in Days of Zip Code Tabulation Area (ZCTA) Floods in the Continental US by ZCTA, January 1, 2008, to December 31, 2017 Regions in white are uninhabited areas.

Geographically, the floods occurred throughout the continental US, with a predominance in the South, Midwest, and Mountain West, with no region unaffected (Figure 1A). ZCTA in the South, Midwest, and Mountain West were more affected by multiple floods. This pattern was more pronounced when visualizing the duration of flooded days (Figure 1B), shown by quintile of flood duration, illustrating a similar geospatial distribution of floods.

### Cohort Analysis

The results of the cohort analysis of all-cause and cause-specific outcomes across the study period for ED visits and hospitalizations are given in Table 2. Across nearly all outcomes except for ED mental health visits, the IRR and 95% CIs demonstrated statistically significant increases in health care use during the flood hazard period compared with the control period. The rate of all-cause ED visits increased by 4.8% (IRR, 1.05; 95% CI, 1.04-1.05), and all-cause hospitalizations increased by 7.4% (IRR, 1.07; 95% CI, 1.07-1.08). The largest IRR increases for ED visits were observed for metabolic and kidney conditions (IRR, 1.08; 95% CI, 1.06-1.11) and injuries (IRR, 1.05; 1.04-1.06). The largest IRR increases for hospitalizations were observed for infectious disease (IRR, 1.12; 95% CI, 1.10-1.13) and metabolic and kidney conditions (IRR, 1.10; 95% CI, 1.07-1.12).

**Table 2.** Zip Code Tabulation Area–Level Regression Analysis by Visit Type and Attributable Risk Percentage and Estimated Attributable Excess Visits for the Preflood and Postflood Periods

Encounter or diagnosis type	Incident rate ratio (95% CI)	Attributable risk, %	No. of visits in flood period	No. of excess attributable visits
**Emergency department visits**				
All cause	1.05 (1.04-1.05)	4.6	462 274	21 265
Cardiovascular	1.05 (1.04-1.06)	4.8	93 697	4497
Metabolic, kidney	1.08 (1.06-1.11)	7.7	20 840	1605
Gastrointestinal	1.03 (1.02-1.05)	3.1	46 884	1453
Infectious disease	1.07 (1.06-1.08)	6.5	67 077	4360
Respiratory	1.04 (1.02-1.05)	3.6	57 908	2085
Injury	1.05 (1.04-1.06)	4.8	76 392	3667
Mental health	1.03 (0.99-1.07)	NA	NA	NA
**Hospitalizations**				
All cause	1.07 (1.07-1.08)	6.9	243 297	16 787
Cardiovascular	1.07 (1.06-1.08)	6.4	59 614	3815
Metabolic, kidney	1.10 (1.07-1.12)	8.7	14 101	1227
Gastrointestinal	1.04 (1.02-1.06)	4.1	21 206	869
Infectious disease	1.12 (1.10-1.13)	10.5	41 674	4376
Respiratory	1.09 (1.07-1.11)	8.3	31 309	2599
Injury	1.08 (1.06-1.11)	7.5	15 690	1177
Mental health	1.09 (1.03-1.14)	7.8	3641	284

**Figure 2.**
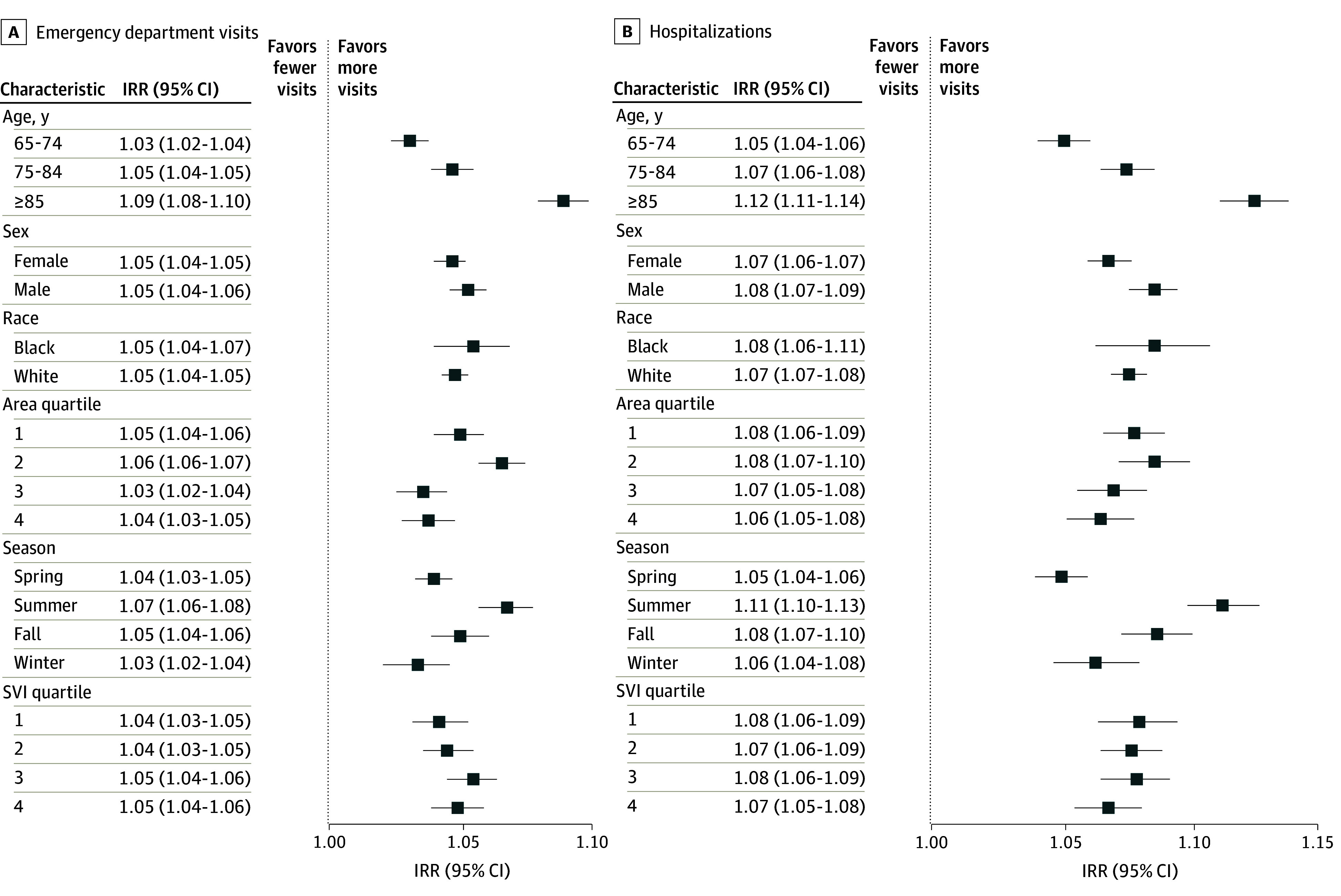
Zip Code Tabulation Area–Level Stratified Regression Analysis Results by Visit Type for the Preflood and Postflood Periods Among the Medicare Beneficiary Cohort Error bars indicate 95% CIs. IRR indicates incident rate ratio; SVI, Social Vulnerability Index.

Attributable risk percentages for ED visits ranged from 3.1% for gastrointestinal diagnoses up to 7.7% for metabolic and kidney conditions. For hospitalizations, 6.4% of cardiovascular admissions and 10.5% of infectious disease admissions were attributable to flood exposure. Excess attributable visits are given in Table 2, with 21 265 excess ED visits and 16 787 hospitalizations attributable to flood exposure. Results of the stratified analysis are shown in Figure 2; eTable 4 in Supplement 1 gives the calculated IRRs and associated 95% CIs as well as attributable risk percentages and excess attributable visits in the study period for all-cause ED visits and hospitalizations. Sex-stratified results revealed an increased rate of ED visits (IRR, 1.05; 95% CI, 1.05-1.06) and hospitalizations (IRR, 1.08; 95% CI, 1.07-1.09) among men compared with women with overlapping CIs. The IRRs increased with age, from 4.9% for hospitalizations among adults aged 65 to 74 years (IRR, 1.05; 95% CI, 1.04-1.06) to 7.3% higher among adults aged 75 to 84 years (IRR, 1.07; 95% CI, 1.06-1.08) and 12.4% greater among adults 85 years or older (IRR, 1.12; 95% CI, 1.11-1.1). The IRRs for ED visits demonstrated a similar trend albeit with a lower magnitude. IRR were also higher among Black compared with White beneficiaries, with overlapping CIs.

Stratified analyses of flood season start demonstrated increased IRRs for ED visits (IRR, 1.07; 95% CI, 1.06-1.0) and hospitalizations (IRR, 1.11; 95% CI, 1.10-1.13) in the summer months, for which the CIs did not overlap with spring but did with other seasons. Neither flood extent nor SVI quartile modified the risk of hospitalizations or ED visits.

### Cost Analysis

The mean ZCTA-level cost was $3230 (95% CI, $3198-$3261) per ED visit and $11 310 (95% CI, $11 252-$11 367) for hospitalizations. The national costs to the Medicare system were estimated to be $69 275 429 (95% CI, $63 010 840-$76 315 210) for ED visits and $191 409 579 (95% CI, $172 782 870-$206 181 300) for hospitalizations (Table 3).

**Table 3.** Cost of Medicare Claims Associated With Postflood Period Compared With Preflood Period^a^

Cost analysis type	No. of excess visits (95% CI)	Cost per visit, mean (95% CI), $	Cost, mean (95% CI), $
Excess emergency department visits	21 265 (19 508-23 627)	3230 (3198-3261)	69 275 429 (63 010 840-76 315 210)
Excess hospitalizations	16 787 (15 277-18 230)	11 310 (11 252-11 367)	191 409 579 (172 782 870-206 181 300)

^a^ All costs have been adjusted to 2017 US dollars.

## Discussion

In this study combining national Medicare claims data with geospatial data on flooding, we found that all-cause ED visits increased by 4.8% and all-cause hospitalizations increased by 7.4% among older adults in communities that experienced a significant flooding event. Our data suggest that during the 10-year study period significant flooding events led to more than 21 265 excess ED visits and 16 787 excess hospitalizations in the Medicare fee-for-service population, amounting to $260 million additional health care spending.

We observed a notable increase in health care encounters after flood exposure for nearly all categories examined, with the highest increases among metabolic and kidney conditions, infectious diseases, injuries, and cardiovascular and respiratory conditions. The attributable risk percentages of these visits ranged from 3.1% to 10.5% of visits, with substantial nationwide excess attributable visits on the order of thousands of visits and a greater volume of ED visits compared with hospitalizations. Mental health diagnoses were the one category not statistically significant for ED visits, although they were for hospitalizations. This finding suggests that Medicare patients experienced additional mental health burdens after floods but may have accessed care through routes other than the ED. Additionally, they may have not been the primary reason for the health care visit but may have been identified as an additional diagnosis during the hospital admission.

Stratified analyses illuminated differential outcomes across various individual-level and flood-specific factors. Men had higher IRRs, consistent with prior literature on sex-specific flood mortality but in contrast with literature on flood morbidity.^2,9^ Age-stratification demonstrated increasing risk with age, consistent with prior literature.^2,9^ Season appeared to modify the association, with summer floods having the greatest association with ED visits and hospitalizations. This finding may be the result of increased risk for bacterial waterborne and foodborne infectious diseases during warmer months.^24^

Other research has focused on floods from specific events, particularly tropical storms. An analysis of ED visits after Tropical Storm Imelda in 2019 in Texas found IRRs for diarrhea (1.15), dehydration (1.07), asthma (1.06), and cardiovascular (1.05) symptoms, consistent with but slightly higher than in our analysis.^25^ That study, like ours, found no difference in sex-stratified results or in results by White or Black racial category stratification. Another storm-related flood analysis of ED visits in New York City from Hurricane Sandy in 2012 found increased ED visit IRRs for cardiovascular (1.10), respiratory (1.35), skin and soft tissue infections (1.20), injuries (1.19), and kidney-related diagnoses (1.44) among adults 65 years or older, primarily in the first week after flood exposure.^26^ These associations are of higher magnitude than those in our analysis, which may be related to their analytic approach of stratification by week, whereas we combined visits during the entire 4-week hazard period, potentially diluting the exposure effect. Notably, the investigators also found the greatest association with kidney diagnoses, suggesting that patients with underlying kidney disease or undergoing dialysis may experience interruptions in receiving routine dialysis maintenance therapy in the setting of flood exposure. Similarly, the other condition-specific results may suggest an increased risk of illness among those with preexisting diabetes, cardiovascular disease, or respiratory disease given the association with health care use for skin and soft tissue infections, cardiovascular disease, and respiratory conditions.

The economic analysis revealed a substantial increase in health care costs associated with flood exposure at the national level. Limited economic assessments of flood exposure have been performed in the US, and the prior focus has been primarily on property damage assessments rather than health impacts and associated health care costs. Despite this, the limited data available suggest that flooding has among the highest per capita health damages of all disasters in the US.^27^ One analysis of 6 climate change–related hazards estimated the costs associated with the Red River flood in North Dakota in 2009, which included costs of premature death ($15.8 million), hospitalization ($839 000), and ED visits ($232 000) calculated from health outcomes of 2 premature deaths, 43 excess hospitalizations, and 263 additional ED visits among an estimated 139 918 exposed people.^27^ The cost analysis we performed was on a different geographic scale, limiting a direct comparison between these results from a particular flood and our national estimates.

Consistent with other work, we found inequities in flood exposures and associated impacts.^28^ Prior work found that these inequities persist in part because relevant federal policies prioritize equality over equity and are insufficient to overcome structural factors that drive risk in certain communities.^29^ In addition, countermeasures known to protect health against floods are not implemented as broadly as needed. These countermeasures include flood prevention through nature-based and structural measures; preparedness through increased awareness, early warning systems, and health system preparedness; and robust response and recovery capacity in the emergency preparedness and health sectors.^30^ Additional attention to implementation of protective measures, enhanced surveillance of health outcomes, and health impact evaluation linking the 2 has the potential to reduce health impacts associated with flooding and improve equity in the distribution of health impacts that remain.

### Limitations

This analysis has limitations. The exposure dataset is limited to floods exceeding the 20-year return period and does not permit a more refined estimation of flood intensity, apart from duration and geographic extent. It is, however, the most extensive geospatial, open-access flood dataset that currently exists. Our study also relies on an ecologic design, and we cannot account for individual beneficiary exposure to the floods, apart from their occurrence within the ZCTA in which the Medicare beneficiary lived. In addition, ZCTAs vary in size, with smaller ZCTAs in urban areas and larger ones in rural regions, which may lead to exposure misclassification if beneficiaries in larger ZCTAs did not directly experience flooding. Such exposure misclassification would bias effect estimates toward the null. Despite this limitation, ZCTAs were chosen because they align with zip code–based patient data and provide a consistent geographic framework during the study period. Limiting to the 4-week period before and after flood start does not allow us to capture delayed health effects. Although our time-series analysis measures changes in acute care use within ZCTAs, which allows regions to serve as their own historical controls, we did not include a contemporaneous control group of unflooded ZCTAs. We limited our analysis to adults 65 years or older who are Medicare beneficiaries, and our findings do not necessarily translate to younger populations or those with commercial insurance.^2,9^ Additionally, the racial composition of the Medicare beneficiaries included in this study reflects the demographics of the flood-affected cohort, who were predominantly from rural areas with a racial distribution differing from the national average. This regional and demographic variability may influence the generalizability of our findings to the broader US Medicare population. Finally, our health care cost estimations used claims data as reported in the Medicare dataset. This approach may not fully capture the total health care costs resulting from flood exposure and therefore may provide a rough estimate of acute care expenditures associated with excess admissions and ED visits rather than a comprehensive simulation or cost evaluation.

## Conclusions

In this cohort study of older adults in the US, large-scale flood events were associated with substantial increases in health care use and spending. The increased rates of ED visits and hospitalizations after floods, particularly among vulnerable groups such as the oldest adults, emphasize the need for targeted public health interventions and enhanced disaster preparedness strategies implemented with attention to reducing inequities in flood-related health impacts. The substantial economic burden observed after such environmental disasters underscores the potential for significantly increased societal costs from unmitigated anthropogenic climate change. Furthermore, this study demonstrated that cost estimates of disasters that do not account for human morbidity and mortality are substantial underestimates. Although our findings provided valuable insights to the understanding of flood-related health impacts, they also highlighted the need for ongoing research in this area, especially in the context of an aging population and a changing climate. Future studies should aim to incorporate more detailed environmental and individual-level data to further refine our understanding of the complex interplay between flood events and population health.

## Notes

Supplement 1. **eAppendix.** Preliminary and exploratory analysis **eTable 1.** 2015 Clinical Classifications Software (CCS) level 3 codes used for the identification of cause-specific outcomes for ZIP code tabulation area (ZCTA)-level cohort analysis **eTable 2.** Frequency of ZIP code tabulation area (ZCTA) floods and median duration in the study period from the Muti-sourced Flood Inventories (MFI) by month **eTable 3.** Frequency of ZIP code tabulation area (ZCTA) floods and median duration in the study period from the Muti-sourced Flood Inventories (MFI) by year **eTable 4.** ZIP code tabulation area (ZCTA)-level regression stratified analysis all-cause incident rate ratio (IRR) and 95% confidence intervals (CI) by visit type and attributable risk percentage (AR%) and estimated attributable excess visits, comparing pre- and post-flood period

Supplement 2.
Data Sharing Statement
