# Supplementary material for: Emergency Care, Hospitalization Rates, and Floods
Source: JAMA Netw Open. 2025 Mar 10;8(3):e250371. doi: 10.1001/jamanetworkopen.2025.0371 (PMC11894485; doi:10.1001/jamanetworkopen.2025.0371)
Supplement: Supplement 1. — eAppendix. Preliminary and exploratory analysis eTable 1. 2015 Clinical Classifications Software (CCS) level 3 codes used for the identification of cause-specific outcomes for ZIP code tabulation area (ZCTA)-level cohort analysis eTable 2. Frequency of ZIP code tabulation area (ZCTA) floods and median duration in the study period from the Muti-sourced Flood Inventories (MFI) by month eTable 3. Frequency of ZIP code tabulation area (ZCTA) floods and median duration in the study period from the Muti-sourced Flood Inventories (MFI) by year eTable 4. ZIP code tabulation area (ZCTA)-level regression stratified analysis all-cause incident rate ratio (IRR) and 95% confidence intervals (CI) by visit type and attributable risk percentage (AR%) and estimated attributable excess visits, comparing pre- and post-flood period [file jamanetwopen-e250371-s001.pdf]

Supplemental Online Content

Wettstein ZS, Parrish C, Sabbatini AK, Rogers MH, Seto E, Hess JJ. Emergency care, hospitalization rates, and floods. *JAMA Netw Open*. Published online March 10, 2025. doi:10.1001/jamanetworkopen.2025.0371

eAppendix. Preliminary and exploratory analysis
eTable 1. 2015 Clinical Classifications Software (CCS) level 3 codes used for the identification of cause-specific outcomes for ZIP code tabulation area (ZCTA)-level cohort analysis
eTable 2. Frequency of ZIP code tabulation area (ZCTA) floods and median duration in the study period from the Muti-sourced Flood Inventories (MFI) by month
eTable 3. Frequency of ZIP code tabulation area (ZCTA) floods and median duration in the study period from the Muti-sourced Flood Inventories (MFI) by year
eTable 4. ZIP code tabulation area (ZCTA)-level regression stratified analysis all-cause incident rate ratio (IRR) and 95% confidence intervals (CI) by visit type and attributable risk percentage (AR%) and estimated attributable excess visits, comparing pre- and post-flood period

This supplemental material has been provided by the authors to give readers additional information about their work.

© 2025 Wettstein ZS et al. *JAMA Network Open*.

eAppendix. Preliminary and exploratory analysis

A preliminary and exploratory analysis was conducted using an interrupted time series analysis approach to explore the population-level impacts and hazard period duration of the flooding and to inform the hazard period selection for the cohort analysis.

An ITSA using a Poisson regression was performed on a range of multi-week periods before and after flood onset across all flooded ZCTA. All-cause ED visits and HA, and certain cause-specific diagnoses for gastrointestinal illness (GI) and mental health (MH) conditions (Appendix 1, Table 1) were aggregated by week and used for the ITSA. The ITSA was performed using 2-, 4-, 6-, and 8-week periods before and after the flood to evaluate the temporal relationship between flood exposure and healthcare utilization. ZCTA-level flood data were used in the analysis, aggregated on a national level. A Poisson regression was used to estimate the relative risk (RR) and 95% confidence interval (CI) of a particular healthcare utilization type in the flood period compared to the pre-flood period.

ITSA model specification for weekly count of healthcare utilization events (Y_T):

$$Log(E(Y_T)) = \beta_0 + \beta_1 Time + \beta_2 Flood + \beta_3 TimeSinceFlood$$

where β_0 represents the baseline level of healthcare utilization, β_1 is the trend of healthcare utilization before the flood start, β_2 is the immediate change in the level of healthcare utilization at the start of the flood, and β_3 is the change in slope after the flood (interaction term).

The ITSA was performed on the 2-, 4-, 6-, and 8-week periods before and after flood start date and 16,536 flooded ZCTA were included in the analysis. The relative risk (RR) and 95% confidence intervals (CI) of healthcare utilization after flood onset, stratified by period duration in weeks, is shown in Appendix 1, Table 2. Across all durations of the ITSA, there was a statistically significant increase in risk of ED visits for all-cause and GI diagnoses of a similar magnitude across durations – for all-cause ED visits at 8 weeks, RR of 1.013 (95%CI: 1.009-1.018). No statistically significant association was seen with inpatient hospitalizations for all-cause or cause-specific diagnoses however, as noted in Appendix 1, Figure 1, the increase in ED visits was noted upon start of the flood while the RR of inpatient admissions demonstrated a trend towards significance during longer hazard period duration in weeks.

The ITSA revealed a statistically significant increase in the population-level risk of ED visits following flood exposure for all-cause, GI-specific, and mental health visits. The ED visit model predictions increased immediately after flood onset; while the model predictions were not statistically significant for hospitalizations, the model predictions demonstrated trends towards increased utilization up to 8 weeks after flood onset. The findings of the ITSA were used to inform the ZCTA-level analysis design of the cohort analysis. *A priori*, the control and hazard period length was 4-weeks in duration and this was supported by the ITSA. We considered using shorter periods, however had concerns about lag times to diagnosis, particularly for mental health conditions and those requiring hospitalization. While considering longer periods greater than 4-weeks, we had concerns about residual confounding and longer secular and seasonal trends and therefore limited the control and hazard periods to 4-weeks in the subsequent ZCTA-level cohort analysis.

© 2025 Wettstein ZS et al. *JAMA Network Open*.

Table 1. 2015 Clinical Classifications Software (CCS) level 3 codes used for the identification of cause-specific outcomes for interrupted time series analysis (ITSA).

Category	*CCS Code*	*Diagnosis*
Mental health (MH)	650	Adjustment disorders
	651	Anxiety disorders
	657	Mood disorders
	659	Schizophrenia and psychotic disorders
	660	Alcohol-related disorders
	661	Substance-related disorders
	662	Suicide and intentional self-injury
Gastrointestinal (GI)	135	Intestinal infection
	144	Enteritis and colitis
	154	Non-infectious gastroenteritis

© 2025 Wettstein ZS et al. *JAMA Network Open*.

Table 2. Interrupted time series analysis (ITSA) regression all-cause and cause-specific relative risk (RR) and 95% confidence interval (CI) results by week and visit type, comparing pre- and post-flood period.

ITSA Duration	*Encounter Type*	*RR*	*Lower 95% CI*	*Upper 95% CI*
2-weeks	ED - All-Cause	1.037	1.026	1.049
	ED - GI	1.120	1.009	1.245
	ED - MH	1.075	1.003	1.153
	HA - All-Cause	1.018	0.993	1.043
	HA - GI	0.982	0.799	1.211
	HA - MH	1.024	0.895	1.172
4-weeks	ED - All-Cause	1.017	1.010	1.024
	ED - GI	1.091	1.025	1.162
	ED - MH	1.029	0.987	1.073
	HA - All-Cause	1.006	0.991	1.020
	HA - GI	0.947	0.836	1.074
	HA - MH	1.045	0.965	1.132
6-weeks	ED - All-Cause	1.011	1.005	1.016
	ED - GI	1.071	1.020	1.126
	ED - MH	1.024	0.990	1.058
	HA - All-Cause	1.000	0.989	1.012
	HA - GI	1.027	0.929	1.135
	HA - MH	1.027	0.964	1.094
8-weeks	ED - All-Cause	1.013	1.009	1.018
	ED - GI	1.065	1.021	1.112
	ED - MH	1.034	1.005	1.064
	HA - All-Cause	1.002	0.992	1.012
	HA - GI	1.062	0.974	1.157
	HA - MH	1.011	0.958	1.068

© 2025 Wettstein ZS et al. *JAMA Network Open*.

Figure 1. Interrupted time series analysis (ITSA) for select diagnoses in the 8-week period before and after flood start. Observed weekly diagnosis counts are shown in red, with ITSA model-predicted values in blue with 95% confidence intervals in grey.

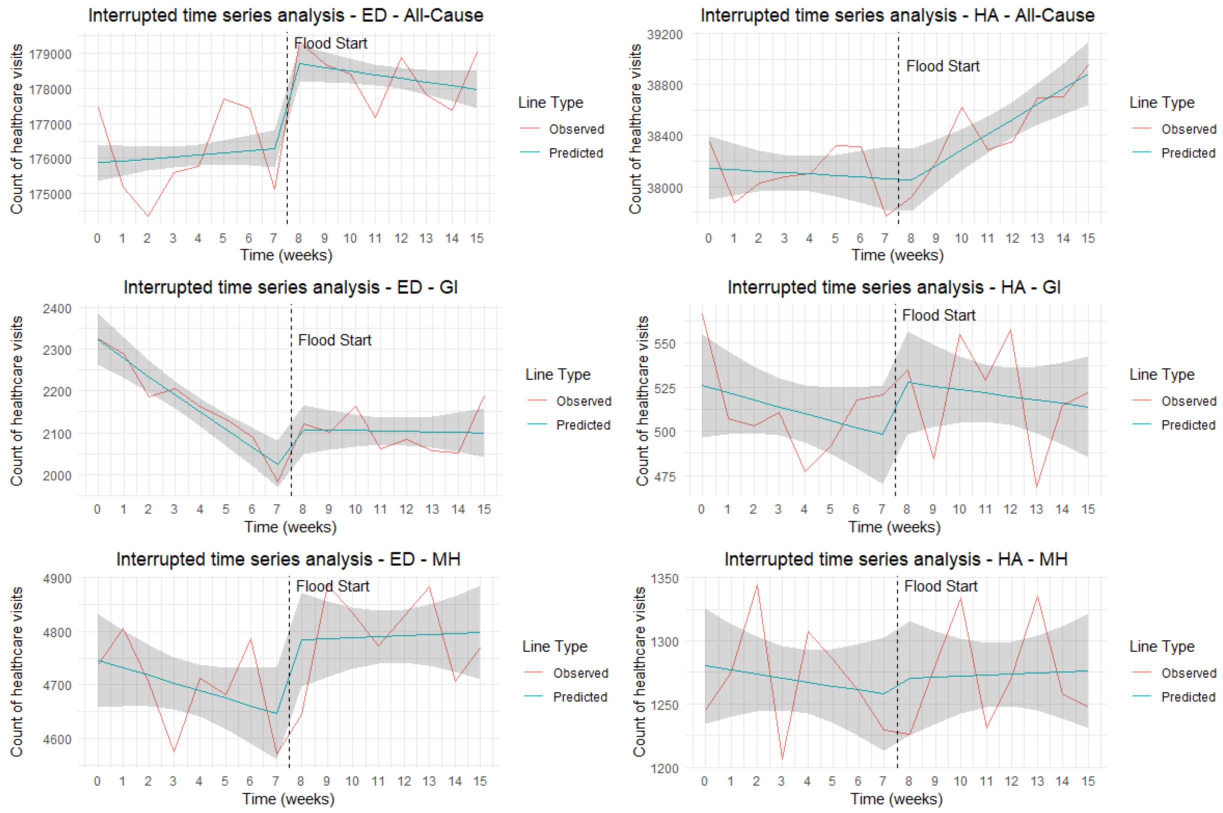

© 2025 Wettstein ZS et al. *JAMA Network Open*.

eTable 1. 2015 Clinical Classifications Software (CCS) level 3 codes used for the identification of cause-specific outcomes for ZIP code tabulation area (ZCTA)-level cohort analysis

Category	*CCS Codes*
Infectious and parasitic diseases (ID)	1-10, 76-78, 90, 92, 122-126, 135, 144, 148, 159, 197, 201
Metabolic, electrolytes, and renal (FEN)	48-52, 55, 58, 156-157
Cardiovascular and cerebrovascular (CV)	96-113, 116-118, 245
Respiratory (Resp)	122-134
Gastrointestinal (GI)	135, 139-140, 142, 144-146, 148-149, 151-155, 250-251
Orthopedic, external injury and poisoning (INJ)	225-244, 2601-2621
Mental health (MH)	650-651, 657-663, 670

eTable 2. Frequency of ZIP code tabulation area (ZCTA) floods and median duration in the study period from the Muti-sourced Flood Inventories (MFI) by month

Month	*Jan*	*Feb*	*Mar*	*Apr*	*May*	*Jun*	*Jul*	*Aug*	*Sep*	*Oct*	*Nov*	*Dec*
ZCTA Floods	476	180	1699	1694	3628	2436	362	1536	1813	920	112	1680
Median Duration (days)	3	22	16	5	40	29	4	9	8	9	9	10

eTable 3. Frequency of ZIP code tabulation area (ZCTA) floods and median duration in the study period from the Muti-sourced Flood Inventories (MFI) by year

Year	*2008*	*2009*	*2010*	*2011*	*2012*	*2013*	*2014*	*2015*	*2016*	*2017*
ZCTA Floods	2051	942	1837	3033	309	975	875	3108	1442	1964
Median Duration (days)	20	7	5	33	11	8	35	11	12	7

© 2025 Wettstein ZS et al. *JAMA Network Open*.

eTable 4. ZIP code tabulation area (ZCTA)-level regression stratified analysis all-cause incident rate ratio (IRR) and 95% confidence intervals (CI) by visit type and attributable risk percentage (AR%) and estimated attributable excess visits, comparing pre- and post-flood period

Encounter Type	*Diagnosis Type*	*IRR*	*Lower 95% CI*	*Upper 95% CI*	*AR%*	*Visits in Flood Period*	*Excess Attributable Visits*
**Sex-stratified**							
Male	ED visits	1.052	1.045	1.059	5.0%	190,121	9,506
	hospitalizations	1.084	1.074	1.093	7.7%	106,703	8,216
Female	ED visits	1.046	1.039	1.051	4.4%	272,153	11,975
	hospitalizations	1.066	1.058	1.075	6.2%	136,594	8,469
**Age-stratified**							
65-74	ED visits	1.03	1.023	1.037	2.9%	193,252	5,604
	hospitalizations	1.049	1.039	1.059	4.7%	99,060	4,656
75-84	ED visits	1.046	1.038	1.054	4.4%	166,206	7,313
	hospitalizations	1.073	1.063	1.084	6.8%	90,461	6,151
85+	ED visits	1.089	1.079	1.099	8.1%	102,816	8,328
	hospitalizations	1.124	1.11	1.138	11.0%	53,776	5,915
**Race-stratified**							
White	ED visits	1.047	1.042	1.052	4.50%	399,640	17,984
	hospitalizations	1.074	1.067	1.081	6.80%	214,567	14,591
Black	ED visits	1.054	1.039	1.068	5.10%	46,851	2,389
	hospitalizations	1.084	1.061	1.106	7.70%	20,984	1,616
**Season-stratified**							
Winter	ED visits	1.033	1.02	1.045	3.2%	64,338	2,059
	hospitalizations	1.061	1.045	1.078	5.8%	34,288	1,989
Spring	ED visits	1.039	1.032	1.046	3.8%	175,027	6,651
	hospitalizations	1.048	1.038	1.058	4.6%	92,562	4,258
Summer	ED visits	1.067	1.056	1.077	6.2%	122,470	7,593
	hospitalizations	1.111	1.097	1.126	10.0%	65,201	6,520
Fall	ED visits	1.049	1.038	1.06	4.7%	100,439	4,721
	hospitalizations	1.085	1.071	1.099	7.8%	51,246	3,997
**Flood area-stratified**							
Quartile 1	ED visits	1.049	1.039	1.058	4.6%	130,759	6,015
	hospitalizations	1.076	1.064	1.088	7.1%	69,697	4,948
Quartile 2	ED visits	1.065	1.056	1.074	6.1%	139,959	8,537
	hospitalizations	1.084	1.07	1.098	7.7%	67,665	5,210
Quartile 3	ED visits	1.035	1.025	1.044	3.3%	100,174	3,306
	hospitalizations	1.068	1.054	1.081	6.4%	54,328	3,477
Quartile 4	ED visits	1.037	1.027	1.047	3.6%	91,382	3,290
	hospitalizations	1.063	1.05	1.076	5.9%	51,607	3,045
**SVI quartile-stratified**							
Quartile 1	ED visits	1.041	1.031	1.052	3.9%	85,750	3,344
	hospitalizations	1.078	1.062	1.093	7.2%	45,743	3,293
Quartile 2	ED visits	1.044	1.035	1.054	4.2%	121,893	5,120
	hospitalizations	1.075	1.063	1.087	7.0%	64,730	4,531
Quartile 3	ED visits	1.054	1.044	1.063	5.1%	130,317	6,646
	hospitalizations	1.077	1.063	1.09	7.1%	68,668	4,875
Quartile 4	ED visits	1.048	1.038	1.058	4.6%	124,314	5,718
	hospitalizations	1.066	1.053	1.079	6.2%	64,156	3,978

© 2025 Wettstein ZS et al. *JAMA Network Open*.
