# Supplementary material for: Emergency Care, Hospitalization Rates, and Floods
Source: JAMA Netw Open. 2025 Mar 10;8(3):e250371. doi: 10.1001/jamanetworkopen.2025.0371 (PMC11894485; doi:10.1001/jamanetworkopen.2025.0371)
Supplement: Supplement 2. — Data Sharing Statement [file jamanetwopen-e250371-s002.pdf]

Data Sharing Statement

Wettstein. Emergency Care, Hospitalization Rates, and Floods. *JAMA Netw Open*. Published March 10, 2025. doi:10.1001/jamanetworkopen.2025.0371

Data
Data available: Yes
Data types: Data (not involving human participants), Data dictionary
How to access data: By request to zwett@uw.edu and upon completion of a data use agreement with the University of Washington.
When available: With publication

Supporting Documents
Document types: Statistical/analytic code
How to access documents: By request to zwett@uw.edu.
When available: With publication

Additional Information
Who can access the data: Researchers whose proposed use of the data has been approved and who have completed a data use agreement with the University of Washington.
Types of analyses: For research.
Mechanisms of data availability: With a signed data use agreement.
